# The Dental Protective Association of the United States

**Published:** 1889-09

**Authors:** 


### THE DENTAL PROTECTIVE ASSOCIATION OF THE UNITED STATES.

A circular setting forth the objects and aims of this organization was mailed three months since to every dentist in the United States whose address could be obtained. If any have not received them they will be mailed upon application. Its first object, as stated in the circular, is to defend the profession against the unjust demands of patentees whose claims are worthless. Let it be distinctly understood that it is *not* the design of the Dental Protective Association to interfere with any man's legitimate business or valid patents, but to stop the enormous abuse of dental patents. Its second object is to bind the dental profession together for mutual protection, strength and helpfulness, with a bank account and without politics.

After collusion had been proved against the old Rubber Company, and the case had been dismissed from the Supreme Court on that account, the dental profession still had to pay royalty to this company because they were powerless to defend themselves *for lack of organization.* Contrast for a moment a body of ten thousand dentists having a bank account of one hundred thousand dollars, with our present helpless condition, "each man for himself, and the devil take the hindermost."

The interest of this fund could be used for scientific investigation, or for any other purpose which would advance the cause of our profession after our patent foes are demolished.

An organization for defense must necessarily differ widely from one for social or political purposes. It must be ready for war at any moment. To insure this, the authority needs to be in the hands of a few. Such an organization is the Dental Protective Association. The power is vested in the directors; the responsibilities are borne by them, and all risks as well. They can sue and be sued, are accountable for the proper handling of the funds, and must take charge of the suit of any member of the Association who is unjustly sued for infringement. Every member, by paying a fee of ten dollars, and assuming a liability of ten more, *only*, (which latter will probably not be needed,) can continue his practice undisturbed, knowing that if sued, he will be furnished with the best of legal talent and evidence, and be relieved of all costs and harassment of suit. It will at once be seen that the largest expenditure of the association must be that of time, energy and thought, on the part of the directors, since there are no salaried officers, and expenses are kept at the minimum.

The first and greatest adversary, though not the only one, with which we have to deal, is the International Tooth Crown Company. Do you know how nearly you have been in its jaws? Look at a list of its patents. On bridge work it has several including the Low, the Richmond and the Sheffield bridges. It has patents on permanent bridges, and patents on removable bridges; a patent on preparing roots for crowns, which includes a patent on freezing the tooth, a patent on cutting off the tooth, a patent on killing the pulp, and on driving it out at the root at the same time (if you can), a patent on filling the end of the root, with material suitable for holding the metallic pin or screw which supports the crown or bridge.

It has even a patent on cement for securing the crown. On crowns it has many patents, including the Beers, or gold crown, the Bitner crown, the Richmond crown and the Sheffield crowns; several of each of the two last named. It has patents on crowns with bands and without bands; patents on crowns secured with screws and without screws; a patent on crowns secured with gutta percha, a patent on crowns secured with cement and a patent on crowns secured with both gutta percha and cement. It has also a patent on crowns covering the ends of roots. In fact, if the validity of these various crown patents owned by the International Tooth Crown Company should be established, it would seem as if all the other crowns now in use would be declared an infringement on that company's patents? Then this company has also a patent on taking impressions, a patent on use of trial plate, a patent on getting articulating models, and a patent on investing the piece for soldering. It has added at least two new patents since our organization was formed, and are continually on the lookout to obtain patents upon which to assert claims against our profession. What is to prevent this company from increasing its patents *ad infinitum*?

Most assuredly its modesty will not stand in the way. It would seem to be a cunningly devised plan on the part of this company to make every man of us pay them tribute in some shape. Can any one doubt that time only is needed, if we do not defend ourselves, to bind us hand and foot? Are we men or slaves, with a profession of fifteen thousand strong, to quietly submit to such an imposition? Our necks are still sore from wearing the yoke imposed by the Rubber Company for many years. Shall we hesitate whether we shall bend them to a heavier one invented substantially by the same class of men?

In answer to questions repeatedly asked we make the following statements. There is no cause whatever for alarm in case the suits now pending in the United States Supreme Court are decided against the dentists. The company confidently expect this decision. We shall not be surprised at it, and are preparing accordingly. If such a decision is rendered, *and you are in the Dental Protective Association*, you have nothing to fear.

The decision of the United States Supreme Court must rest upon the evidence it now has as shown by its record. But this does not prevent a new trial of these claims in the United States Supreme Court, or any other Federal Court, by the Protective Association upon another record, and we are confidently assured by our attorneys that we have abundant evidence to prove the utter worthlessness of these patent claims.

Suits instituted and won against individual dentists outside the Protective Association will not affect it in the least. No individual member can procure the evidence, or can afford the time and money necessary to compete with a great monopoly like the International Tooth Crown Company. Besides it is easy to see how a suit could be conducted against an individual favorable to the interests of the company, and thus have a repetition of the old Rubber Company's methods and results, for we are dealing practically with the same set.

Some have hesitated to join the DENTAL PROTECTIVE ASSOCIATION for fear it might not succeed, and that they would be liable to greater exactions on the part of our opponents. This fear is groundless for the law does not allow the company to charge one man more than another when they have a royalty already fixed:

The methods of this company in securing licensees are peculiar, to say at least. Its manager enters a town or city scares all the dentists possible into taking out a license, and then advertises in the local papers that these licensees are the only dentists in the place entitled to use crown and bridge work under their patents, and that all others, as well as persons wearing crowns and bridges made by other parties, are liable for damages. No person wearing a crown or bridge made under any process or method patent whatever can be made liable for damage. This process of intimidation is not confined to individual dentists, but letters threatening suits for damage to their business from the publication of our last circular, have been sent to our treasurer, Mr. Lyman L. Gage, who was in no way responsible for the circular, also to each director of the DENTAL PROTECTIVE ASSOCIATION, and to the editors of some of our leading dental journals for aiding us in this attempt to defend ourselves. Do you think the time has come for *concerted action*? It is the one thing which our enemy fears. Not a member of the DENTAL PROTECTIVE ASSOCIATION has yet been sued. Will you come in and help us to put down the hydra-headed monster that would devour our living, or will you wait until victory is ours and then cry, "Betsey and I killed the bear?" It was wisdom for any man to settle with the International Tooth Crown Company upon their own terms, odious as they were before we had an organization. It is folly to do so now. We have evidence enough to defend any member of our Association in any suit that may be brought against him on any patent now threatened by the I. T. C. Co

A few things should be well borne in mind: First, that in joining the Association you are getting a great amount of protection for a very small amount of money.

The least you can do to even matters up is to aid the Chairman in securing other members. His services are given gratuitously, and his time is as valuable as your own. Let every member feel a *personal* responsibility in this matter. You can also aid by securing additional evidence, the more the better if it is good. We desire any one having evidence, relating to either crown or bridge work, done before the year 1877, to send to us a detailed description of such work, enclosing a drawing or sketch of the work when completed, if possible, no matter how roughly such sketch may be made, and giving the name of the party for whom such work was made, and for how long a time used; send also any ancient dental books which refer to crown or bridge work of a permanent character.

Please sign, if you have not already done so, the enclosed By-Laws, sending name and address, *plainly written*, with membership fee, *without delay*, to J. N. Crouse chairman Board of Directors of the DENTAL PROTECTIVE ASSOCIATION, 2231 Prairie Avenue, Chicago Ill., who will forward you receipt.

Messrs. Offield & Towle are exclusive patent counsel in all our matters, and will take charge of any case against any member when reported to the Association. Their address is 185 Dearborn Street, (Adams Express Building), Chicago, Illinois. They will take pleasure in answering any interrogatories as to these matters of any member of the Association.

Remember, the DENTAL PROTECTIVE ASSOCIATION only guarantees to take care of members who join before suit has been commenced against them. Remember that we are fighting for our freedom and rights—nothing more—nothing less In the words of one of the nation's early patriots:

"*Blandishments will not fascinate us, nor will threats of a "halter" intimidate. For, under God, we are determined that, wheresoever, whensoever, or howsoever we shall be called to make our exit, we will die free men.*"

